# Community Mental Health Services in Andean Peru: Mapping Supply and Demand

**DOI:** 10.3390/ijerph23040512

**Published:** 2026-04-16

**Authors:** Milagros Alvarado, Daniel Mäusezahl, Stella Hartinger, Andrea Fernandez-Rodriguez, Maria Melero-Dominguez, Francisco Diez-Canseco, Günther Fink, Ricardo Peña-Sánchez, Irene Falgas-Bague

**Affiliations:** 1 Swiss Tropical and Public Health Institute, 4123 Allschwil, Switzerland; milagros.alvarado@unibas.ch (M.A.); stella.hartinger.p@upch.pe (S.H.); guenther.fink@swisstph.ch (G.F.); irene.falgasbague@swisstph.ch (I.F.-B.); 2 University of Basel, 4003 Basel, Switzerland; 3 Center of Climate Change and Health (CLIMA), Universidad Peruana Cayetano Heredia, Lima 150135, Peru; 4 Institute of Social and Preventive Medicine, 3012 Bern, Switzerland; andrea.ferro.0211@gmail.com; 5 Graduate School for Health Sciences, University of Bern, 3012 Bern, Switzerland; 6 Human Services Psychology Program, University of Maryland, Baltimore County, Baltimore, MD 21250, USA; mmelero1@umbc.edu; 7 Universidad Científica del Sur, Lima 150142, Peru; fdiezcanseco@cientifica.edu.pe; 8 CRONICAS Center of Excellence in Chronic Diseases, Universidad Peruana Cayetano Heredia, Lima 150135, Peru; 9 Ministerio de Salud del Perú, Lima 15072, Peru; calopenax@gmail.com

**Keywords:** mental health, health system reform, access to care, community-based care, Peru

## Abstract

**Highlights:**

**Public health relevance—How does this work relate to a public health issue?**

- Mental health service access remains uneven for rural populations despite national health system reforms in Peru;
- Fragmented service delivery and weak referral pathways shift care responsibility onto users and families, delaying treatment.

**Public health significance—Why is this work of significance to public health?**

- It provides empirical evidence on the implementation of community mental health reforms in rural and resource-constrained contexts;
- It highlights the role of cultural, social, and health system factors in shaping access to mental health care.

**Public health implications—What are the key implications or messages for practitioners, policy makers and/or researchers in public health?**

- Strengthening workforce stability, referral pathways, and culturally responsive care is critical to sustain rural mental health services.
- Structured and supervised collaboration with community networks and traditional healers (task-sharing) can enhance referrals, treatment adherence, and early detection, provided clear clinical boundaries and referral safeguards are maintained.

**Abstract:**

Peru’s recent national mental health (MH) reforms aim to decentralise care and expand access to MH services for rural populations by integrating services into primary healthcare through the expansion of Community Mental Health Centres (CMHCs). Evidence on the implementation of these reforms at the local level remains limited. This qualitative study aimed to (i) describe the structure and implementation framework of MH services, (ii) analyse local understandings of MH; and (iii) examine pathways to care and identify barriers and facilitators to MH service implementation from both the supply (service providers) and demand (users and community members) perspectives. MH services were mapped across three provinces of northern Peru using a review of national MH policies, 2 focus group discussions, and 31 semi-structured interviews. Data were analysed thematically to explore local understandings of MH, pathways to care, and health system barriers. Local understandings of MH are shaped by cultural beliefs, social norms, and economic conditions, with many individuals experiencing distress initially relying on family networks or traditional healers. Stigma and expectations of a quick recovery hinder engagement with formal services. While the expansion of CMHCs has improved geographical access to specialised care in rural areas through proximity and being patient-centred, the implementation of respectful provider interactions remains uneven. Weak referral pathways and limited coordination between primary care centres and CMHCs frequently shift the responsibility for navigating care onto users and their families. Family involvement and culturally sensitive practices foster trust and support continued engagement. Persistent challenges include the limited capacity of service providers, high staff turnover, and the follow-up mechanisms, stigma, and tensions between cultural and biomedical understandings of MH. Peru’s expansion of CMHCs represents a significant health system reform to improve equitable access for rural populations. To sustain these gains, it will be necessary to strengthen workforce stability, clarify referral processes, and integrate culturally responsive approaches within primary care systems, offering lessons for similar resource-constrained contexts.

## 1. Introduction

Since the 2022 World Mental Health Report [1], mental health (MH) has gained global attention as a critical public health concern [2]. In Peru, MH care was historically concentrated in tertiary care and limited to three psychiatric hospitals in Lima [3,4]. A gradual shift toward integrated and decentralised services began with the 2004 “Guidelines for Action in Mental Health” and was reinforced by the 2012 “General Health Law”, which recognised the rights of people with MH conditions and promoted universal, cost-free access to care [5]. In 2018, the community MH model was formally implemented, integrating the screening, diagnosis, and treatment of common mental disorders and psychosocial disabilities into Primary Care Centres (PCCs), and establishing Community Mental Health Centres (CMHCs), with 291 centres now operating nationwide [6,7,8].

According to this reform, PCCs are intended to improve access to MH care by ensuring the sufficient availability of MH providers, as well as an adequate supply of psychotropic medication throughout the country. However, in practice, many PCCs, particularly in rural areas, face persistent shortages of both specialised staff and medications [3]. CMHCs implement the national MH policy, providing care from the prevention to treatment of severe illness. They also aim to play a critical role in supporting PCCs through managing patients with mild to moderate conditions that do not require a referral to specialised MH hospitals. Multidisciplinary teams staffing the CMHCs aim to provide psychotherapy, rehabilitation, and medication management for individuals with various MH conditions [4]. Each centre is expected to include at least one psychiatrist, psychologist, and nursing staff [9]. However, staffing varies widely, especially in rural areas. In 2020, Peru had 3.5 psychiatrists per 100,000 people, with 80% located in Lima, while Cajamarca has only 0.2 psychiatrists and 9 psychologists per 100,000 inhabitants [10].

Despite these policy developments, it remains unclear how reforms translate into truly accessible services [11]. Although MH is formally recognised as a national priority, gaps persist in implementation, coverage, and sustained support, particularly in rural areas [12].

In this study, rural areas refer to communities located outside major urban centres, typically characterised by low population density, geographic dispersion, and limited access to specialised health services. Rural contexts in Peru are heterogeneous across the highlands, Amazon, and coastal regions; this study focuses specifically on Andean highland communities, which present distinct geographic barriers, socio-economic conditions, and cultural belief systems that shape their health-seeking behaviour and interaction with formal health services. Cajamarca communities are largely engaged in agriculture, commerce, and mining, and are embedded in rich cultural traditions that influence their understandings of health and MH. These characteristics are particularly relevant in the context of Peru’s ongoing MH reform.

This study examines MH care in Andean communities within the context of ongoing MH policy reform. Specifically, it aims to (i) describe the structure and implementation framework of MH services, (ii) analyse local understandings of MH, and (iii) examine pathways to care and identify barriers and facilitators to MH service implementation from both the supply (providers) and demand (users and community members) perspectives. Against this background, this study examines MH reform implementation in Andean communities, linking national policy objectives with service delivery realities in rural areas.

## 2. Materials and Methods

### 2.1. Setting

The study was conducted in the provinces of Cajamarca, Cajabamba, and San Marcos in the Cajamarca region, in the northern highlands of Peru, between 1800 and 3900 metres above sea level. These provinces include both urban and rural communities. The livelihood of this population primarily depends on agriculture, commerce, construction, and mining [13,14].

The Cajamarca region was selected as the empirical study site due to its relevance for examining implementation of Peru’s MH reform in a highland rural context. Longstanding health systems research collaborations in high-altitude rural communities in the Cajamarca region, including prior work on environmental health and chronic disease prevention [15,16,17]. This ongoing engagement facilitated contextual understanding, and established community relationships and logistical feasibility for qualitative data collection.

### 2.2. Study Design

This qualitative study addressed three objectives. First, to describe the structure and implementation framework of MH services, we reviewed national policy documents, including policy guidelines and subnational plans, and conducted MH professionals’ interviews to understand how primary care practitioners perceive MH implementation within the broader healthcare system. Second and third, to analyse local understandings of MH and examine pathways to care including barriers and facilitators to implementation, we conducted focus group discussions (FGDs) and in-depth interviews with adults. The demand side included community members and individuals with lived experience of MH symptoms, referred to as *users.* The supply side comprised MH professionals (e.g., psychologist, nurses, and psychiatrists) involved in delivering MH services, from the formal public sector, referred to as *practitioners*.

### 2.3. Participants and Recruitment

Participants were recruited across the Cajamarca region using tailored approaches for each subgroup to ensure diversity in perspectives and experiences. Demand-side participants included the following: (I) community members who were 18 years or older, lived in the study area, and could offer insights into community perspectives and attitudes towards MH, recruited via local leaders, and (II) individuals with lived experience of MH conditions, previously enrolled in two ongoing research projects—a multigenerational cohort study and a randomised control trial including adults with MH symptoms and parents of young children—and classified as likely having mild-to-moderate depression, anxiety, or stress symptoms using the Depression, Anxiety, and Stress Scale (DASS-21). Supply-side participants included professionals and were drawn from one PCC and three CMHCs. Further recruitment followed a snowball sampling approach.

In total, 42 participants took part in the study. The demand side comprised 18 participants: 11 community members who participated in two focus group discussions (5 women, 6 men), and 7 female service users who participated in individual interviews. The supply side comprised 24 practitioners, all of whom participated in individual interviews (18 women, 6 men). Participants were recruited from both rural and urban communities across the three provinces, as detailed in Table 1.

### 2.4. Instruments, Data Collection, and Procedures

Two separate interview guides were developed: one for supply-side interviews (practitioners), and one for demand-side interviews (users). A third guide was developed for focus group discussions (FGDs). All guides were reviewed by psychological and psychiatric experts and covered system-level factors, experiences, and care pathways. The interviews and FGD guides are provided in Supplementary File S1 (Tables S1–S3).

All interviews and FGDs were conducted in Spanish, in person, by the first author. Each interview and FGD lasted 45–60 min and was audio-recorded with participants’ consent. Data collection continued until thematic saturation was reached.

### 2.5. Analysis

Interviews and focus groups were transcribed, anonymised, and imported into MAXQDA 24 for analysis [18]. All transcripts, including FGD records and relevant national policy documents, were coded following the same analytical process to integrate documentary insights. We applied a reflexive thematic analysis approach that combined both inductive and deductive strategies using Andersen’s Behavioural Model of Health Services as an initial coding framework for analysis [19]. Codes were developed using a single unified codebook across both individual interview and focus group transcripts, and themes were refined through iterative comparison of perspectives across data sources to identify convergent and divergent patterns (Supplementary File S1). To ensure reliability, three coders (all native Spanish speakers) independently reviewed a subset of transcripts. Discrepancies were resolved by consensus, and intercoder agreement was assessed to ensure consistency. Detailed information about the methods is provided in Supplementary File S1.

## 3. Results

### 3.1. The Status of the Health System and MH Integration in Peru

**Figure 1:**
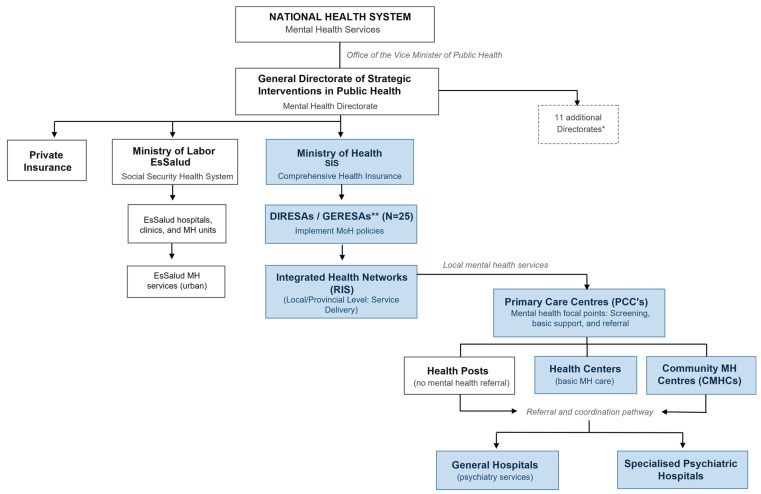
Structure of the mental health service delivery within the Peruvian health system. * The General Directorate oversees 12 directorates in total; this chart highlights the Mental Health Directorate. ** DIRESA: Direccion Regional de Salud; GERESA: Gerencia Regional de Salud.

The document review showed Peru’s health system is fragmented with overlapping public and private providers. The Ministry of Health (MoH) provides care to most of the population, especially those without formal employment, through its regional health directorates (DIRESA/GERESA) (coverage: ~62%). EsSalud, supervised by the Ministry of Labor and Employment Promotion, covers formally employed individuals through the social security system (coverage: ~26%). Other branches of the public sector serve specific groups, such as members of the police and armed forces. In parallel, a growing private sector serves those able to afford private insurance or out-of-pocket care (coverage: ~8%) [12]. Figure 1 provides an overview of how the delivery of MH services is organised within the broader health system.

A document analysis also showed that MH services are unevenly distributed, with MoH playing a regulatory role in designing, monitoring, and supervising national MH reforms while the operation and delivery of local MH services are the responsibility of subnational governments, which may face challenges in adequately managing resources allocated to primary care services. The MoH has driven the integration of MH into primary care and expanding CMHCs, whereas EsSalud and other subsystems also provide MH care but with considerable variation in reach and integration within the broader health system [4,12].

The Peruvian MH reform was formalised through National Law No. 29889 (2012), declaring MH care a national priority and mandating its integration into primary health services [20]. This was reinforced by Law No. 30947 (2019), defining MH as a universal right and emphasising a person-centred, community-based care model. The 2018 Mental Health Policy Guidelines established five areas of strategic implementation [9]. These areas were operationalised through the National Plan for Strengthening CMHCs (2018–2021) [21]. MH services follow a stepped-care model, which distributes care across three levels: PCCs handle early detection, mild conditions, and basic care; CMHCs provide specialised outpatient services via interdisciplinary teams and act as referral points; and psychiatric hospitals manage severe inpatient cases, focusing on reducing hospital dependence. Practitioners reported that the coordination with health centres, health posts, and CMHCs is often weak, with referral pathways unclear or underutilised.

Practitioner interviews and previous studies highlighted persistent resource constraints. Despite the policy push, the availability of trained MH professionals remains uneven, particularly in rural areas. The supply of psychotropic medications is limited, a pattern that is similarly observed for other noncommunicable diseases [22]. These gaps continue to constrain the treatment of MH conditions [10].

#### 3.1.1. Local Implementation Challenges in Community Mental Health Centres

Interviews at three CMHCs in the study area confirmed these challenges. Practitioners reported centres were not always fully staffed, with psychiatrists sometimes shared between facilities, and shortages of other essential professionals (e.g., occupational and speech therapists). Additionally, some staff were employed on short-term contracts, limiting the continuity of care and contributing to service instability.

*“We’re even lacking more professionals here, for example, there’s no speech therapist available; we’re missing professionals.”*

(Practitioner, 30 years old; interview)

Practitioners also highlighted infrastructure challenges that undermine service quality and community trust. Many CMHCs occupy repurposed facilities originally belonging to other ministries, former community centres, or public spaces (e.g., multipurpose halls where local sports and cultural activities were held, or former health posts no longer in operation). This has caused frustration among residents who feel they have lost important community gathering spaces. Facilities are often under-resourced, lacking private rooms, equipment, and space for individual or group therapy, limiting their ability to meet the demand.

*“When the Ministry of Transport donated the land to the CMHCs, some people tried to claim ownership. The community even argued vehemently, saying, ‘No, this is for crazy people, we’re not crazy!’”*

(Practitioner, 40 years old; interview)

#### 3.1.2. Referral Pathways and Interconnected Care

According to national MH care guidelines and policy documents, the referral process follows a stepped-care model. In this model, the identification and initial management of MH conditions begins at PCCs, where practitioners screen for symptoms and provide basic care. When the symptoms are moderate to severe or exceed the scope of primary care, patients are referred to CMHCs for evaluation and treatment [9,23]. Policy documents specify that CMHCs should coordinate with complementary services, including (I) protected homes and halfway houses for severe or chronic cases requiring transitional support; (II) psychosocial rehabilitation centres to promote social skills and community reintegration; and (III) occupational rehabilitation centres providing vocational training, guidance, and employment support.

For more complex cases requiring intensive psychiatric treatment, CMHCs refer individuals to specialised MH general hospitals with psychiatric units [24]. However, interviews conducted in Cajamarca reveal gaps between policy and practice. While protected homes exist, psychosocial and occupational rehabilitation centres are still absent, limiting CMHCs’ ability to implement the intended interconnected care model. Practitioners also highlighted that referral pathways between health centres, posts, and CMHCs are often unclear or inconsistently applied, contributing to fragmented care and delays in access.

*“Lower-level centres are supposed to refer users when they identify moderate or severe problems. But that happens in only a small percentage of cases. Most of the time, it’s the users themselves or their relatives who come here because someone else was already receiving care.”*

(Practitioner, 30 years old; interview)

**Figure 2:**
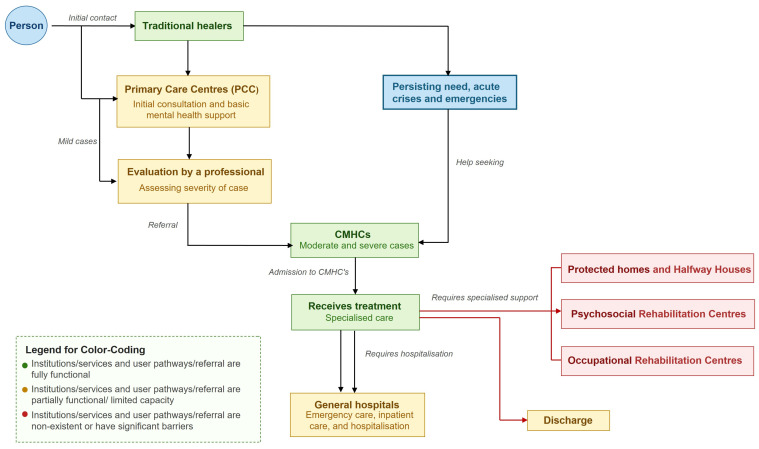
Usage and referral pathway, and user’s journey in the mental health services in Cajamarca.

Figure 2 illustrates the actual user journey and referral pathways in Cajamarca, showing entry points, initial contacts, and the subsequent referral steps across PCCs, CMHCs, and other services. Some users may discontinue at various stages, though this attrition is not explicitly shown in the figure.

### 3.2. Participant Characteristics

All 42 participants were Spanish-speaking and resided in three provinces of Cajamarca region. Among users, the women were older on average, had a lower educational attainment (including no formal schooling), and were more often homemakers or unemployed, whereas the men more frequently had technical or university education and were employed or students. The practitioners were predominantly women. They comprised mainly psychologists and nurses, some family doctors, a psychiatrist, a social worker, and a pharmaceutical chemist. Most were employed in CMHCs (See Table 2).

#### 3.2.1. Local Journeys Through Mental Health Care

The following sections present the findings from the interviews and FGDs. The results are organised around four main themes emerging from the qualitative analysis: (1) perceptions and recognition of mental health conditions, (2) navigating formal and informal care networks, (3) perception of care, and (4) areas of improvement (See Table 3).

##### Perceptions and Recognition of Mental Health Conditions

Practitioners reported that emotional distress is often normalised as a temporary response to life’s challenges rather than a reason to seek care. Users reported that they often express distress through physical symptoms or behaviours such as headaches, irritability, or withdrawal, which can obscure the recognition of MH conditions. Severe symptoms like hearing voices may be attributed to supernatural causes, prompting individuals to seek traditional healers

*“When you go to church, they ‘heal’ your mental health. They might say, ‘You’re sad, so go to church, they’ll pray for you, and you’ll be healed.’ Sometimes it works, and sometimes it doesn’t.”*

(User, 38 years old; interview)

*“They believe it’s a curse or a spell. So, they go to “curanderos” or use medicinal plants.”*

(Practitioner, 46 years old; interview)

Stigma remains a significant barrier. Practitioners noted formal health services are still strongly associated with severe illness or “madness”, which discourages early help-seeking. This scepticism is particularly strong among older adults, who often view psychological support as unnecessary, preferring to manage symptoms with medication. Gender norms also shape MH-related perceptions and behaviours. Many women reported being raised to endure domestic violence, framed as part of marital duty, which can limit their ability to recognise and seek support for the MH consequences of such experiences. Men are expected to appear strong and unemotional, suppressing their expression of distress, and contributing to under-reported and untreated MH conditions. Broader social factors also affect MH expression, with economic hardship, poor living conditions, and substance use (especially alcohol) frequently linked to family stress, violence, and MH struggles.

*“Sometimes there isn’t enough even for the week.”*

*“When I tell my children I can’t give them something, they take it badly, they go away being sad too.”*

(User, 35 years old; interview)

Religious beliefs and the involvement of traditional healers (“curanderos” [1]) play important roles in how distress is understood and addressed before people access formal MH services. Practitioners and users noted that it is common to consult traditional healers. Spiritual explanations often provide the first line of attribution, and advertisements for traditional healer services are common in public spaces. Users also rely on over-the-counter “emotional” remedies to self-manage symptoms.

*“If I argue with my dad and then get a headache, I know it’s from being upset, I take my syrup and it goes away.”*

(User, 29 years old; FG)

Social support networks and personal coping strategies play key roles in managing MH difficulties. Users reported that close family members and friends often provide primary emotional support, helping them cope before or alongside professional care. Spiritual practices or self-help activities (e.g., gardening, cooking, or going for walks) further contribute to resilience in the face of adversity. Last, practitioners noted gender differences in help-seeking behaviours, with women being more likely than men to seek help.

Overall, cultural beliefs, stigma, gender norms, economic hardship, and social relationships shape how MH is understood and addressed.

##### Navigating Formal and Informal Care Networks

Users and practitioners mentioned that the first challenge lies in the formal health system itself: long waiting times, shortages of specialised personnel, and rigid scheduling, which not only slow access but also reduce the perceived quality of care, particularly for those traveling from remote areas. Practitioners also noted gaps in public knowledge about MH services’ availability, function, and target.

*“Well, the only thing I know is that they give you help for when you’re feeling depressed …that’s the only thing they offer, nothing else.”*

(User, 37 years old; interview)

Within this limited context, practitioners employ adaptive strategies such as flexible scheduling, immediate emotional support (e.g., earliest appointment or prioritising urgent cases), and coordination with institutions like schools and health posts to enhance access and responsiveness.

*“We have people who come from far away… and a strength here, is adjust our schedules so that person can come, and maybe it takes them 3 h, but they get…I don’t know, occupational therapy, the doctor, and the psychologist.”*

(Practitioner, 37 years old; interview)

Practitioners and users identify that social stigma often poses a deeper and more enduring obstacle than structural barriers. Fear of judgment, shame, and community gossip deter many from seeking care, even when services are available.

*“Some people might feel embarrassed, right? They don’t want to talk about their things. They feel ashamed and think maybe it’ll be told to someone else.”*

(User, 20 years old; interview)

*“In small towns everyone knows each other, they start saying maybe she got treated there, that something’s wrong, that she’s crazy… in smaller places, it’s like, I won’t go because maybe they’ll talk about what I have… so the gossip is very strong because of that.”*

(Practitioner, 40 years old; interview)

Practitioners described working alongside traditional healers as a daily clinical reality. Rather than confronting or dismissing traditional practices, most adopted a non-confrontational stance, positioning formal care as an additional option rather than a replacement. When parallel use was detected, that is, when users were simultaneously attending traditional healers and the CMHC, practitioners generally tolerated this, provided that the clinical treatment remained uninterrupted.

*“If they go in parallel, there’s no problem, because they already have their treatment here, and their beliefs too.”*

(Practitioner, 35 years old; interview)

Some practitioners reported actively integrating culturally accepted practices into their clinical approach, for instance, validating the use of medicinal plants with a recognised scientific basis alongside prescribed pharmacological treatment, or adapting therapeutic language to align with patients’ belief systems. However, practitioners also identified a critical referral threshold: when traditional healing delayed or replaced formal care entirely, particularly in cases of psychosis or severe disorders, consequences could be serious.

*“When the patient is completely deteriorated, hearing voices… that’s when they finally come, because the curanderos didn’t heal them.”*

(Practitioner, 36 years old; interview)

Overall, navigating care involves moving between formal and informal systems shaped by structural barriers, stigma, and cultural beliefs. While practitioners adopted adaptive strategies to bridge these gaps, informal and traditional networks remain a primary and often more accessible first point of support.

##### Enablers and Barriers of Access to Care

The above-mentioned structural barriers constrained access. Users frequently travelled a long distance, facing transportation costs, only to find no available staff, creating frustration and discouraging continued attendance. Practitioners confirmed that shortages of specialist’s limit service capacity contribute to treatment discontinuation, compounded by limited professional training opportunities.

*“Discharge due to discontinuation is the most frequent now. One of the problems causing abandonment is that, unfortunately, we have very limited specialised human resources for such a large population.”*

(Practitioner, 37 years old; interview)

Practitioners adopted adaptative strategies to sustain engagement, including reminder calls or combining multiple appointments into one visit, which proved effective. While telemedicine expanded access for some, poor connectivity limited its effectiveness in rural areas. Nonetheless, follow-up phone calls and flexible scheduling helped maintain engagement for users with low adherence and/or severe disorders.

*“They’re entered a Continuity of Care Program, where visits can even be every other day… through phone calls, regular follow-ups. It’s more continuous monitoring when the patient has low adherence, low support… especially for those with a diagnosis of severe mental disorder.”*

(Practitioner, 38 years old; interview)

Practitioners consistently described respectful, compassionate care contributing to treatment adherence, even when logistical difficulties arose. Cultural sensitivity further strengthened these ties. For example, rather than rejecting traditional practices, practitioners acknowledged their role in the healing process, using psychoeducation to explain how psychopharmacological treatment could complement traditional practices.

*“We always try to validate their beliefs, like if a certain herb helped them, but we also explain the importance of the prescribed treatment, right? We’ve been able to do this with some populations.”*

(Practitioner, 31 years old; interview)

Practitioners and users noted that personal expectations, community narratives, and relationships shape engagement in care. Many enter treatment influenced by personal beliefs, or second-hand accounts, sometimes carrying hopeful expectations based on stories of recovery and positive outcomes observed in others.

*“The woman I saw in the market is now much better, and my mother-in-law also told me it helped my brother, he improved in school and in his behaviour. And so did my little brother.”*

(User, 20 years old; interview)

On the other hand, concerns about being judged or mistreated are common, often rooted in community stigma that frames MH services as relevant only for extreme or “abnormal” cases. However, when families understand that improving a relative’s MH benefitted the entire household, the stigma is softened, and their involvement increases.

*“Before the mental health centre opened, people in the area were afraid… they’d say, ‘No, there’ll be crazy people.’ No matter how much information you give them, they still think mental health equals ‘crazy”*

(User, 37 years old; FG)

In fact, family support emerged as a key enabler of the continuity of care, facilitating transport, medication access, and adherence. However, families themselves also faced barriers, such as distance, poverty, or conflicting obligations. In such cases, some patients were left to navigate services alone, often leading to premature disengagement. When families were actively engaged, they played essential roles in administering medication and monitoring adherence, requiring ongoing guidance from health staff.

*“There are remote areas where we identify cases, but they don’t come. They say it’s because they can’t afford it… Sometimes we send the medication with a family member.”*

(Practitioner, 37 years old; interview)

Overall, while structural limitations posed significant challenges, respectful provider interactions, family involvement, and adaptive practices supported continued engagement.

##### Pathways for Policy Implementation and Improvement

Practitioners highlighted several key areas for improving the MH system in Cajamarca, focusing on infrastructure, accessibility, public awareness, and workforce capacity. Strengthening the first level of care emerged as a priority. Although health centres and posts are responsible for identifying cases and referring individuals, practitioners noted that its potential remains unrealised, leading CMHCs to assume additional roles such as outreach and early case identification.

*“If they identify cases (PPCs), they’re supposed to refer them, but they rarely send us anything. So, we end up doing the outreach and identification, which isn’t actually our role.”*

(Practitioner, 40 years old; interview)

Facility infrastructure was another area frequently mentioned. Practitioners emphasised the importance of permanent, purpose-designed facilities to improve service quality and build community trust. Operating CMHCs in rented or repurposed spaces may hinder efforts to raise awareness and establish a reliable presence. They also highlighted the need for residential or transitional facilities to support individuals experiencing homelessness or domestic violence. While protected homes exist within the Cajamarca area, access remains limited.

Human resources were seen as central to service improvement. Practitioners reported persistent shortages of specialised professionals, especially psychiatrists, and suggested strategies to improve recruitment and retention in care, including flexible employment arrangements and revising salary structures to reflect the workload and responsibilities.

*“We have gaps in psychiatry; we just can’t provide that care…psychiatrists have left because the pay is low. Other places offer better salaries, so they leave”*

(Practitioner, 38 years old; interview)

Improving public awareness through ongoing educational campaigns was seen as essential to reduce stigma and increase community engagement. Practitioners emphasised outreach in schools and community spaces to correct misconceptions and encourage early help-seeking.

Finally, practitioners expressed the need for ongoing professional development, with more regular and inclusive training supported by adjusted schedules and budgets without disrupting service delivery.

*“We ask to come visit us, to spend time here, but so far, we haven’t been given the opportunity. It would be a chance to observe and learn how things are handled in other centres, especially in Lima, where things are more developed.”*

(Practitioner, 39 years old; interview)

## 4. Discussion

This study examined MH systems in three provinces of Cajamarca, describing the structure and implementation framework of MH services, analysing local understandings of MH, and identifying barriers and facilitators to implementation from the practitioners’ and users’ perspectives. The findings highlight the interplay between policy implementation and cultural, social, and structural factors shaping MH care in the northern highlands. Peru’s MH reform represents a major policy shift from hospital-based psychiatry to decentralised, community-oriented care, exemplified by the rapid expansion of 12 CMHCs in Cajamarca, bringing specialised services closer to rural populations. Peru stands out in Latin America for the pace and scale of its national MH reform [25,26].

Despite a strong policy framework promoting community MH care integrated into primary care, implementation appears to remain uneven due to structural and socio-cultural constraints in the region. Many PCCs lack MH professionals and face a high staff turnover, limiting early detection and timely care [27,28,29]. The weak coordination between PCCs and CMHCs compounds these gaps, with informal, delayed, or absent referrals placing the burden of care navigation on users and families. Without clear pathways or follow-up, users and families frequently assume the responsibility of identifying appropriate services, seeking out CMHCs independently, and doing so with limited information about what specialised services exist, where they are located, or how to access them when needed. This delays access and undermines the reform’s goal of an integrated, stepped-care approach. These coordination challenges are compounded by a broader system fragmentation. Each subsystem (MoH, EsSalud, and others) operates with its own administrative and budgetary framework, limiting coordinated regional planning and resource allocation. This separation can result in the duplication of services in urban areas while leaving rural populations with limited alternatives. As a result, regional health authorities face restricted flexibility in reallocating staff and financial resources across subsystems, complicating efforts to implement the reform coherently. These challenges highlight the importance of effective health system planning and coordination in translating national policy into practical, local-level outcomes.

Our findings reveal that many individuals turn to culturally familiar systems before accessing formal MH care. Peruvian traditional medicine is rooted in cosmological beliefs that view illness as a disruption in the balance between body and soul, often linked to one’s environment and community. Restoring this harmony is the domain of healers, whose rituals and practices, transmitted across generations, remain widely used [30]. This cultural understanding of health continues to shape how people interpret and respond to emotional distress. Our findings reveal how this belief systems influence the lived experiences of MH care, particularly in the ways users navigate the formal system. Such perspectives often stand in contrast to the clinical approach dominant within the formal MH system and its staff, where practitioners emphasise the importance of diagnosis, long-term treatment, and multidisciplinary care, potentially reducing adherence [31]. When practitioners adopt culturally sensitive approaches, acknowledging traditional beliefs, engaging family networks, and using psychoeducation to explain how medical and traditional practices can coexist, trust and participation tend to increase. Yet, this coexistence is not without tension. Our findings show that practitioners work to find a balance: respecting the parallel use of traditional healers when it does not interfere with treatment, while remaining alert to situations where the exclusive reliance on traditional healers delays access to formal care, particularly in severe cases. This points to the need for clearer community-level mechanisms to identify and redirect cases where traditional care alone is not sufficient. In rural settings especially, these strategies were seen to foster more meaningful engagement and bridge the gap between diverse understandings of MH and healing. This blending of clinical and traditional practices reflects cultural continuity and practical necessity.

Our findings show that stigma remains a significant barrier to early help-seeking in Cajamarca, operating not only at the individual level but also through community dynamics and gender norms that normalise suffering and discourage help-seeking. This multilevel nature of stigma, spanning personal shame, community gossip, and cultural expectations, helps explain why structural improvements alone are insufficient to increase service uptake. This finding aligns with previous research showing that stigma constrains the reach of preventive and community-based interventions [32]. Gender dynamics further shape these patterns differently for men and women: while women in our study faced stigma tied to social roles, men’s reluctance to seek help likely reflects expectations to appear strong and unemotional, contributing to under-reported and untreated conditions. However, the absence of male voices in individual interviews limits our understanding of gender-specific barriers for men. Given that masculine norms in rural Peru discourage expressing distress, men may face distinct barriers to help-seeking that our data does not fully capture. Tackling these barriers requires efforts at multiple levels: collaborations between CMHCs, schools, and community leaders help mitigate these by compensating for systemic gaps, fostering trust and encouraging engagement. This localised approach reflects both practitioner commitment and the need to adapt MH services to the social realities.

Our findings highlight that these localised efforts operate within persistent structural constraints. Geographic, economic, and infrastructural barriers, alongside system fragmentation, workforce shortages, and limited investment in primary-level care, continue to exacerbate disparities across the Peruvian health system. While our study did not explore the discontinuation of care and treatment in depth, practitioners’ accounts suggest that both logistical and cultural barriers contribute. Logistical factors, including distance, transport costs, and limited appointment availability due to workforce shortages, were frequently cited as drivers of treatment abandonment. Cultural factors, such as a preference for traditional healers’ services or a low perceived need for continued care, also played a role, often intersecting with logistical ones. Addressing discontinuation will therefore require targeted strategies that tackle both dimensions simultaneously. Similar challenges are observed in the management of other chronic conditions, where resource constraints and fragmented coordination shift responsibility onto individual practitioners and local networks, often reinforcing rather than reducing inequities [33,34]. Leveraging digital technologies, such as phone-based support, and telepsychiatry presents opportunities to strengthen decentralised MH services and support policy implementation in resource-limited settings [35,36,37].

Building on the identified challenges, practitioners’ proposals for improving the MH system are essential, even with resource constraints. These include (I) enhancing the first level of care through targeted training and supervision for existing staff, (II) improving referral processes with clearer protocols and better communication between facilities, and (III) expanding public awareness and community engagement using low-cost digital or community-based campaigns. Such strategies not only address the immediate service gaps but also provide evidence-informed guidance for regional and national policymaking, with potential relevance across similar LMIC settings. Even organisational initiatives can yield meaningful improvements if coordinated across multiple health system levels. Their success depends on optimising resource and financial management and securing sustained support throughout the health system.

This study provides valuable insights from the perspectives of community members and MH practitioners working on the frontlines of Peru’s reform efforts, particularly in rural areas. The findings highlight both the advances following policy shifts and how users and providers navigate formal and informal care networks. However, regional variations beyond Cajamarca and informal practices in other cultural contexts were not explored. The absence of input from policymakers and subnational programme managers limits our understanding of policy formulation and implementation, including the effects of resource management on local service delivery. Additionally, while access-related barriers to care were identified, the study did not explore in depth the specific circumstances driving the discontinuation of care. The relatively small sample limits generalisability but is appropriate for in-depth, exploratory qualitative research.

## 5. Conclusions

Several priorities emerge. At the community level, training community health agents, implementing contact-based stigma reduction, and integrating traditional healers as intercultural MH agents can improve early identification and engagement with formal care. At the workforce level, flexible training models and mentoring can strengthen capacity. At the system level, clearer referral protocols linking PCCs and CMHCs are essential to reduce fragmentation and improve timely access to care.

Peru’s expansion of CMHCs has improved geographical access to specialised care in rural areas; yet, workforce instability and weak coordination continue to constrain their impact. Cultural beliefs and stigma shape help-seeking in ways that structural expansion alone cannot address. Sustaining reform gains requires strengthening referral integration and embedding culturally responsive approaches within primary care. Expanding services is necessary, but building the conditions for those services to reach and retain the people who need them most is what turns reform into impact.

## Figures and Tables

**Table 1:** Distribution of study participants by data collection instrument.

Group	Focus Group Discussions (N = 2)		Interviews (N = 31)	
	**Females**	**Males**	**Females**	**Males**
Demand side: Users	5	6	7	-
Supply side: Practitioners	-	-	18	6

**Table 2:** Demographic characteristics—mental health service users and practitioners.

Characteristics		Female	Male
Demand side—Users		N = 12	N = 6
Mean age in years		36	31
Formal education attainment (level completed)			
	No formal education	3	0
	Primary school	1	0
	Secondary school	3	2
	Higher education (technical or university)	5	4
Occupation			
	Self-employed	2	0
	Employee	2	4
	Student	1	2
	Home-maker	7	0
Place of residence (urbanicity)			
	Rural	9	6
	Urban	3	0
Supply side—Practitioners		N = 18	N = 6
Mean age in years		35	41
Role			
	Psychologists	7	3
	Nurses	8	1
	Family Doctor	1	1
	Psychiatrists	0	1
	Social Worker	1	0
	Pharmaceutical chemist	1	0
Type of facility			
	Community MH centre	17	6
	Health centre	1	0

Demand side—Users: community members (FGDs, *n* = 11) and individuals with lived experience (interviews, *n* = 7). Supply side—Practitioners: mental health professionals from the formal public sector (interviews, *n* = 24).

**Table 3:** Themes from interviews and FGDs with users and providers on MH experiences in Cajamarca, Peru.

Theme	Subtheme	Description
Perceptions and recognition of mental health conditions	Mental health beliefs	Distress is often seen as a normal part of life rather than a condition requiring formal care.
	Perceived causes	Distress is frequently attributed to supernatural causes, family problems, or economic hardship.
	Support system	Family and friends are typically the first source of support, alongside spiritual practices and personal coping strategies (e.g., gardening or walking).
	Social cultural factors	Gender norms, poverty, and community relationships shape how distress is expressed and managed.
	Role of traditional healers	Traditional healers are often consulted prior to formal MH services, and over-the-counter emotional remedies are used to self-manage symptoms.
Navigating formal and informal care networks	Barriers to access	Staff shortages, long distances, long waiting times, rigid scheduling, and limited public awareness hinder access to care.
	Enablers to access	Flexible scheduling, coordination with schools and health posts, and immediate emotional support facilitate entry into formal MH services.
	Role of stigma in access to care	Fear of judgment and community gossip discourage help-seeking even when services are available.
Perception of care	Expectation of care	Community narratives and personal beliefs influence expectation of formal services.
	Barriers to retention in care	Staff shortages and logistical difficulties lead to treatment discontinuation.
	Enablers to retention in care	Culturally sensitive care, follow-up contact, flexible scheduling, family involvement support, *and* positive recovery narratives support continued engagement with services.
Areas of improvements	Awareness campaigns	Community outreach and education are needed to reduce stigma and encourage early help-seeking.
	Professional training	Flexible and continuous training opportunities are needed to strengthen workforce capacity.
	Primary care centres	Enhancing first-level care for early identification, management, and referral of MH conditions is a priority.

## Data Availability

The data supporting the findings of this study are not publicly available to protect participant confidentiality. Data may be available from the corresponding author upon reasonable request.

## Supplementary Materials

The following supporting information can be downloaded at https://www.mdpi.com/article/10.3390/ijerph23040512/s1: Extended methods (study setting, study design, participants and recruitment, instruments, data collection and procedures, and analysis), and supplementary tables: Table S1. User’s focus group guide—Excerpt of interview questions illustrating key domains, Table S2. User’s interview guide—Excerpt interview questions illustrating key domains, Table S3. Practitioner’s interview guide—Excerpt interview questions illustrating key domains.

## Abbreviations

The following abbreviations are used in this manuscript:

MH	Mental Health
CMHCs	Community Mental Health Centres
PCCs	Primary Care Centres
FGDs	Focus Group Discussions
MoH	Ministry of Health
