# Supplementary material for: Community Mental Health Services in Andean Peru: Mapping Supply and Demand
Source: Int J Environ Res Public Health. 2026 Apr 16;23(4):512. doi: 10.3390/ijerph23040512 (PMC13116352; doi:10.3390/ijerph23040512)
Supplement: Supplementary file 1 [file ijerph-23-00512-s001.zip › Supplementary File S1.pdf]

Supplementary file S1:

Table of Contents

Project Summary 2

Methods (Extended version) 2

Setting 2

Study design 3

Participants and recruitment 3

Instruments, Data collection and Procedures 4

Analysis 5

Supplementary tables 6

Project Summary

This qualitative study examines how mental health care is organised, accessed, and perceived in rural and peri-urban communities in northern Peru. Conducted in three provinces of the Cajamarca region, the research situates mental health within a broader social, geographic, and health system context characterised by limited resources and diverse community structures. Specifically, the study aimed to: (i) describe the structure and implementation framework of mental health services, (ii) analyse local understandings of mental health, and (iii) examine pathways to care and identify barriers and facilitators to mental health service implementation from both supply (providers) and demand (users and community members) perspectives.

Using qualitative methods, the study incorporated perspectives from community members, individuals with lived experience of mental health symptoms, and practitioners involved in mental health service delivery. Policy and planning documents were reviewed alongside primary qualitative data to support a system-level understanding of mental health care. Data were analysed thematically using an established health services framework.

By focusing on contextual factors shaping mental health care in rural Andean settings, this study provides background insight to inform research, policy, and practice in similar low-resource contexts.

Methods (Extended version)

Setting

The study was conducted in in the provinces of Cajamarca, Cajabamba and San Marcos in the Cajamarca region, in the northern highlands of Peru between 1800 and 3900 metres above sea level (MASL). These provinces include both peri-urban and rural communities. The population depends on agriculture and livestock for their livelihood, with additional employment in commerce, education, health, construction, municipal work, and mining [1]. Educational attainment remains low, with only 17.9% of the population over the age of 15 having completed higher education, and an illiteracy rate of 14.8%, well above the national average. In terms of religious affiliation, Catholicism has traditionally dominated, although evangelical and other protestant denominations have grown in recent decades. [2]

The Cajamarca region was selected as the empirical study site due to its relevance for examining implementation of Peru's MH reform in a highland rural context. Longstanding health systems research collaborations in high-altitude rural communities in the Cajamarca region, including prior work on environmental health and chronic disease prevention [3-5]. This ongoing engagement facilitated contextual understanding, established community relationships, and logistical feasibility for qualitative data collection.

Study design

This qualitative study, we mapped the current landscape of mental health services in rural and peri-urban communities of Cajamarca. To provide an understanding of the mental health system in the study context, we first mapped the existing structures and services through a combination of document analysis and MH professionals' interviews. National policy documents were reviewed and used to describe how mental health is positioned within the broader healthcare system. The first section of the results presents this overview based on both documentary and participant accounts.

We employed focus group discussions and in-depth interviews with adults (aged 18 and above) to explore perceptions, experiences, challenges, and gaps in the use and provision of mental health services. This approach allowed us to capture diverse perspectives from both service users and practitioners, helping to identify barriers to access, structural limitations, and community-level needs within the mental health system. We collected data from two main study groups, defined by their relationship to the mental health system.

A. The demand side: included individuals with a current or a potential need for mental health care, namely community members and individuals with lived experience of mental health symptoms.
B. The supply side: comprised mental health professionals and other care practitioners involved in delivering mental health services in the MH care formal sector.

Identification of participants for the demand-side group was supported by two ongoing projects being conducted. From a multigenerational cohort, we could identify pregnant women and their family members with mental health conditions [Blinded for review], and from a randomised control trial [Blinded for review] where we identified parents of young children presenting mental health symptoms. Both subgroups represent vulnerable rural populations exposed to poverty and harsh high-altitude environments, caregiving responsibilities and limited social support that contribute to their mental health status

The demand side included community members and individuals with lived experience of MH symptoms, referred to as users. The supply side comprised MH professionals (e.g., psychologist, nurses, psychiatrists) involved in delivering MH services, from the formal public sector, referred to as practitioners.

Participants and recruitment

Participants were recruited across the three provinces of Cajamarca using tailored approaches for each subgroup to ensure diversity in perspectives and experiences.

A. Demand side participants:
    a. Community members from urban and rural communities without necessarily having a history of mental health conditions, but who could offer insights into the community perspective and attitude towards mental health in the area. Recruitment was supported by local leaders following a snowball sampling

approach. Two focus groups were conducted with 11 participants, ensuring representation across geography and gender.

b. Individuals with lived experience of mental health conditions - previously enrolled in the multigenerational cohort or a randomised control trial projects and scored for mild-to-moderate depression, anxiety or stress symptoms using the Depression, Anxiety, and Stress Scale (DASS-21). Individuals with severe symptomatology or medical conditions that could interfere with participation were excluded from this study. Although both men and women were sought for this subgroup, only women were available and agreed to participate at the time of recruitment. This group provided insight into the lived experience of mental health challenges within the current care system A total of 7 in-depth interviews was conducted at the participants houses.

B. Supply-side participants: This group included professionals involved in planning, coordination, and delivering of mental health services at local levels (e.g., psychologist, nurses, psychiatrists). Participants were drawn from a range of institutions, such as PCC's and CMHCs. Recruitment followed a top-down snowball sampling approach, and individuals were invited to participate if they held a position within their institution and had knowledge or experience in the organisation or provision of mental health services. A total of 24 in-depth interviews were conducted with supply-side practitioners at the facilities. During focus groups, one field assistant occasionally facilitated organization but did not participate in discussions

Instruments, Data collection and Procedures

Interviews and focus groups discussions (FGDs) were conducted in Spanish, in person, by the the first author, lasting 45-60 minutes, and were audiotaped with participants consent

Interviews and FGDs followed a predetermined topic guide. Separate topic guides were developed for each participant group (users and practitioners) to reflect their specific experiences and roles within the mental health system. Each guide was semi-structured and included open-ended questions aimed at exploring participants' perceptions and experiences in depth. While the guides were tailored to each group, key thematic areas were consistently explored across interviews and FGDs, included participants lived experiences, cultural beliefs, attitudes toward mental health care, help-seeking behaviour, perceptions of the health system, barriers and facilitators to access, and how care-related decisions are made.

The guide for supply-side participants focused on system-level factors, including organisational structure, resource availability, and coordination between services, policy implementation, and practitioners' perspectives on community needs. All guides were reviewed and commented on by psychological and psychiatrist experts. Interviews and focus groups discussions were conducted in person.

Data collection continued until thematic saturation was reached (n=42 participants). This approach ensured sufficient depth and diversity of perspectives across both demand- and supply-side participant groups, supporting the study's analytical rigor and comprehensiveness.

Table 1. Distribution of study participants by data collection instrument

Group	Focus group discussions (N=2)		Interviews (N=31)	
	Females	Males	Females	Males
Users	5	6	7	-
Practitioners	-	-	18	6

Analysis

Researchers' audio-recorded the interviews and focus groups and fieldnotes were taken. Interviews were transcribed in vernacular Spanish. Once transcription was completed, all transcripts were manually reviewed to remove any identifying information before being imported into MAXQDA24 for analysis, [6] ensuring participant confidentiality. Alongside interview and focus group transcripts, relevant national policy documents and government materials were also uploaded into the software and coded to integrate documentary insights into the analysis.

We applied a reflexive thematic analysis approach that combined both, inductive and deductive strategies. An initial coding framework was established from the topic guide, which was formulated based on Andersen's Behavioural Model of Health Services as a guiding framework.[7] Inductive coding allowed us to capture new themes that emerged organically from the participants' narratives, ensuring that unanticipated insights were integrated into the analysis.

The coding process began with the selection of relevant codes, followed by the development of clear definitions for each. These codes were then organised into broader themes, with representative quotes attached to illustrate key patterns in the data. Transcripts were not returned to participants for comment or correction and did not provide feedback on the findings. However, to enhance reliability, three coders (all native Spanish speakers) independently reviewed a subset of transcripts. Any discrepancies were discussed and resolved through consensus, and an intercoder agreement assessment was conducted to ensure consistency in thematic interpretation.

Supplementary tables

***Table S1**. User's focus group guide – Excerpt of interview questions illustrating key domains*

Questions

1. What comes to mind when you hear the term "mental health"? Do people talk about their mental health issues to their friends? Or to their family members? To their work colleagues? For example, do they talk about feeling sad or stressed, or do they keep it to themselves?
2. What do you think stops people from asking for help when they are feeling bad?
3. How do you think people in your community react when someone talks about feeling sad or worried?
4. Do you know any traditional ways your community deals with feeling sad or worried? Can you give an example? Probes: *mal del colera* liquid
5. What are the services that are currently available for people struggling with mental health issues in your community? Can you mention it? Do you think these places help people who are struggling with their feelings?
6. Can you think of anything that makes it easier or difficult for people in your community to get help when they are feeling sad or worried? What do you think we could do to help more people in your community get help when they need it?

***Table S2**. User's interview guide - Excerpt interview questions illustrating key domains*

Questions

7. What comes to mind when you hear the term "mental health"?
8. How do people in your community talk about mental health conditions? with friends, family, or colleagues? For example, do they share feelings of sadness or stress, or do they tend to keep it private?
9. Do you think it is acceptable to ask for help when feeling sad or worried? What might prevent people from seeking help?
10. Are there any traditional practices in your community for coping with sadness or worry? Can you give an example?
11. Do you have access to information or services related to mental health? What support is currently available in your community?
12. What factors make it easier or harder for people in your community to access mental health support? (probes: family support, distance, cultural beliefs, stigma, financial barriers)

Table S3*. Practitioner's interview guide - Excerpt interview questions illustrating key domains*

Questions
1. Can you tell me what your experience is working in mental health? Can you tell me about the roles and responsibilities in the health facility/CMHC's since you took up this position?
2. What are major mental health issues you see in the country/community? Where do people go for mental health care? Can you describe the pathway that people follow in your area when accessing the mental health system?
3. What happens to a person accessing the service? What are the processes followed in the mental health facilities?
4. What makes it easy for community members in the country/community to participate in these programs/ services? What makes it challenging for community members in the country/community to participate in these programs/services?
5. What do you see as the greatest challenges around improving mental health in this setting?
6. Thinking about the future, if you could do one thing to improve the mental health of the community members, what would it be?

References

1. Hartinger SM, Nuño N, Hattendorf J, Verastegui H, Karlen W, Ortiz M, et al. A factorial cluster-randomised controlled trial combining home-environmental and early child development interventions to improve child health and development: rationale, trial design and baseline findings. BMC Med Res Methodol. 2020;20(1):73.10.1186/s12874-020-00950-y
2. Instituto Nacional de Estadistica e Informatica. Compendio estadistico 2024-Cajamarca. 2024.
3. Hartinger SM, Lanata CF, Hattendorf J, Verastegui H, Gil AI, Wolf J, et al. Improving household air, drinking water and hygiene in rural Peru: a community-randomized-controlled trial of an integrated environmental home-based intervention package to improve child health. Int J Epidemiol. 2016;45(6):2089-99.10.1093/ije/dyw242
4. Nuño N, Mäusezahl D, Hattendorf J, Verastegui H, Ortiz M, Hartinger SM. Effectiveness of a home-environmental intervention package and an early child development intervention on child health and development in high-altitude rural communities in the Peruvian Andes: a cluster-randomised controlled trial. Infectious Diseases of Poverty. 2022;11(1):66.10.1186/s40249-022-00985-x
5. Sanchez-Samaniego G, Hartinger SM, Tallman PS, Mäusezahl D. Cardiovascular Disease in the Peruvian Andes: Local Perceptions, Barriers, and Paths to Preventing Chronic Diseases in the Cajamarca Region. International Journal of Public Health. 2021;Volume 66 - 2021.10.3389/ijph.2021.1604117
6. Software V. MAXQDA 24. Berlin: VERBI Software; 2024.
7. Alkhawaldeh A, M AL, Rayan A, Abdalrahim A, Musa A, Eshah N, et al. Application and Use of Andersen's Behavioral Model as Theoretical Framework: A Systematic Literature Review from 2012-2021. Iran J Public Health. 2023;52(7):1346-54.10.18502/ijph.v52i7.13236
